# First Report on the Seroprevalence and Risk Factors Associated with *Toxocara* Infection in Blood Donors from Romania

**DOI:** 10.3390/pathogens14090857

**Published:** 2025-08-29

**Authors:** Ana Alexandra Ardelean, Rodica Lighezan, Sorin Ursoniu, Sergiu Adrian Sprintar, Daniela Adriana Oatis, Alin Gabriel Mihu, Maria Alina Lupu, Tudor Rareș Olariu

**Affiliations:** 1 Discipline of Parasitology, Department of Infectious Diseases, Victor Babes University of Medicine and Pharmacy, 300041 Timisoara, Romania; paduraru.ana@umft.ro (A.A.A.); lighezan.rodica@umft.ro (R.L.); rolariu@umft.ro (T.R.O.); 2 Center for Diagnosis and Study of Parasitic Diseases, Department of Infectious Disease, Victor Babes University of Medicine and Pharmacy, 300041 Timisoara, Romania; sergiu.sprintar@umft.ro (S.A.S.); daniela.oatis@umft.ro (D.A.O.); 3 Patogen Preventia, 300124 Timisoara, Romania; 4 Clinical Laboratory, Municipal Clinical Emergency Hospital, 300254 Timisoara, Romania; 5 Regional Blood Transfusion Center, 300737 Timisoara, Romania; 6 Discipline of Public Health, Department of Functional Sciences, Victor Babes University of Medicine and Pharmacy, 300173 Timisoara, Romania; sursoniu@umft.ro; 7 Center for Translational Research and Systems Medicine, Department III Functional Sciences, Victor Babes University of Medicine and Pharmacy, 300173 Timisoara, Romania; 8 Aurel Ardelean Institute of Life Sciences, Vasile Goldis Western University of Arad, 310414 Arad, Romania; 9 Department of Biology and Life Science, Faculty of Medicine, Vasile Goldis Western University of Arad, 310025 Arad, Romania; 10 Clinical Laboratory, Institute of Cardiovascular Diseases, 300310 Timisoara, Romania

**Keywords:** epidemiology, zoonotic infections, toxocariasis, ELISA, cross-sectional study, immunoglobulin G

## Abstract

Human toxocariasis is a neglected tropical disease with a potentially major impact on public health. Our aim was to assess the seroprevalence and risk factors associated with *Toxocara* seroprevalence in blood donors from Romania. Serum samples were obtained from 1347 Romanian blood donors and serologically tested for anti-*Toxocara* antibodies. An epidemiological questionnaire was used to determine the risk factors associated with *Toxocara* infection. The overall prevalence of *Toxocara* antibodies was 29.6%, with a significant age-associated increase (*p* < 0.001). A higher rate was observed in individuals from rural areas compared to urban areas (*p* = 0.002) and in males compared to females (*p* = 0.001). In univariate statistical analysis, seropositivity was significantly associated with household ownership (*p* < 0.001), contact with soil (*p* < 0.001), owning dogs (*p* < 0.001), cats (*p* = 0.003), and consumption of undercooked poultry (*p* = 0.002). In a stepwise multivariate logistic regression model, only a lower level of education, age, male gender, consumption of undercooked or raw poultry, and contact with soil were associated with higher *Toxocara* seroprevalence. Our findings suggest a significant prevalence of *Toxocara* infection in this region. The identified risk factors highlight the necessity of health education programs that focus on public awareness and promote preventive behaviors, especially among at-risk populations.

## 1. Introduction

Toxocariasis is a zoonotic disease caused by roundworms from the genus *Toxocara* [1]. The only species belonging to the *Toxocara* genus known to cause infection in humans are *Toxocara canis* and *Toxocara cati* [2]. However, most cases of human toxocariasis are caused by *T. canis* [3].

*Toxocara* infection is one of the most common helminthic zoonoses in the world, with an estimated 1.4 billion affected individuals, primarily in tropical and subtropical areas [4]. The highest rates were registered in the Marshall Islands, St. Lucia, and La Reunion, where rates reached up to 87%, 87%, and 93%, respectively [5]. High prevalences were associated with poor socioeconomic status and low environmental status; these factors may worsen in warm, humid conditions like those seen in tropical regions [6].

The life cycle of *Toxocara* species can be either direct, involving a single host, or indirect, involving multiple hosts [7]. The definitive hosts for *T. canis* and *T. cati* are domestic dogs and cats, respectively [1]. Up to 200,000 eggs are laid daily by female worms; these eggs are passed in the feces of dogs and cats and will embryonate in the environment in about 2–3 weeks. Due to their remarkable resistance, infectious eggs can remain viable for years [8,9]. Livestock serve as crucial paratenic hosts [6]. Potential paratenic hosts of *Toxocara* include rodents, birds, rabbits, cattle, sheep, pigs, and poultry [3]. Humans are considered accidental hosts and become infected through ingestion of raw vegetables, water, soil contaminated with embryonated eggs, or by consuming raw meat from paratenic hosts contaminated with *Toxocara* larvae [5]. After ingestion of the eggs, infectious larvae hatch but are not able to develop into adult worms [10]. Instead, they pass through the intestinal wall, enter the circulation, and migrate throughout the body to various organs. Depending on the organ involved, larvae trigger a significant inflammatory response as well as a variety of clinical symptoms [11]. Host immune responses target the migrating larvae, leading to increased cytokine and antibody production along with local inflammation and eosinophilia [12]. A significant percentage of larvae go into hypobiosis, or a hibernation state, in which they can remain for several years. Other larvae become trapped inside granulomas and are further eliminated by the host’s immune system in response to soluble larval antigens of an excretory–secretory origin [13].

Although *Toxocara* infection in humans is usually asymptomatic, in some individuals, it may cause a variety of clinical manifestations, including cough, fever, wheezing in visceral toxocariasis, and eye redness, even vision loss in ocular toxocariasis [14,15]. The influence of the disease on medicine and public health may be underestimated because of its non-specific symptoms [11,12]. However, there are four clinical types of toxocariasis (visceral larva migrans, ocular larva migrans, covert toxocariasis, and neurotoxocariasis) that might have a negative impact on human health [11,12].

*Toxocara* infection is diagnosed through serology, along with imaging techniques to find encapsulated larvae in tissues [16]. For accurate diagnosis, corroboration of the clinical symptoms with laboratory analyses (eosinophils and total IgE) should be performed [17]. Human toxocariasis seroprevalence surveys have traditionally utilized the Western blot (WB) analysis and enzyme-linked immunosorbent assays (ELISAs) [5]. A positive serologic test result suggests that a patient might have been exposed to polluted environments and might be at risk for developing clinical symptoms [18]. Since *Toxocara* larvae may live for ten years in the human body and their migration or death releases antigens continuously, stimulating the formation of antibodies, *Toxocara* antibody titers are not utilized to assess the efficacy of therapy [18].

Various seroepidemiological studies evaluated the global seroprevalence of *Toxocara* antibodies in the human population. The seroprevalence of *Toxocara* antibodies varies between countries, from 3.7% in Slovakia [19] to 15.6% in Estonia [20] and 22.5% in Serbia [21,22]. The estimated worldwide seroprevalence of infection in humans is 19.0% [1].

Limited data are available to the international medical community regarding the seroepidemiology of human toxocariasis in Romania. The existing publications include studies conducted on small, convenient samples that assessed serologic tests recorded in hospitalized patients or individuals referred to a private laboratory [23,24].

To our knowledge, there are no scientific studies regarding the seroepidemiology of *Toxocara* infection in Romanian blood donors. Therefore, the objective of this study was to determine for the first time the seroprevalence of anti-*Toxocara* antibodies and the risk factors associated with the seroprevalence in healthy blood donors from Western Romania.

## 2. Materials and Methods

**Figure 1:**
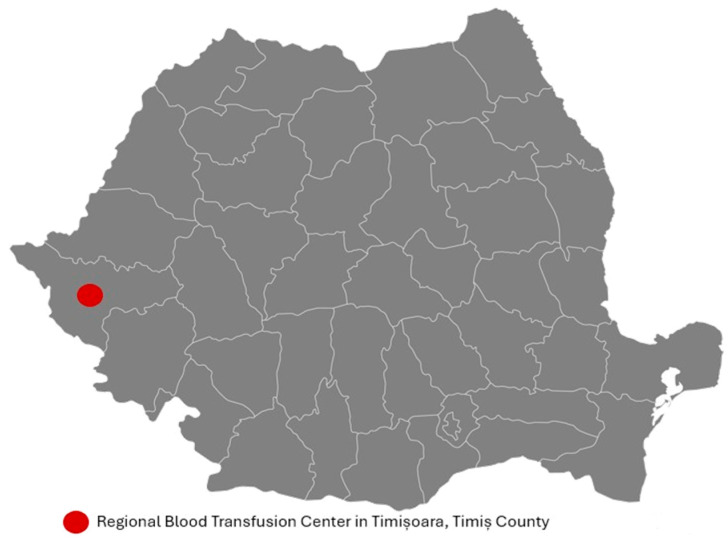
Geographical location of the study area: Timiș County, Romania.

We performed a cross-sectional study on 1347 consecutive blood donors who attended the Regional Blood Transfusion Center in Timișoara, Romania, during 19 November–21 December 2018 (Figure 1). The study comprised all blood donors who presented at the transfusion center during the specified period, met the Romanian Ministry of Health’s donation eligibility requirements, and agreed to participate in the survey [25]. The blood donation procedure was not permitted for participants with type I diabetes, schizophrenia, epilepsy, chronic hepatitis, liver cirrhosis, HIV, cancer, anemia, or asthma [25].

Venous blood samples (5 mL) were collected from each survey participant. The sera were kept at −20 °C until they were analyzed at Victor Babes University of Medicine and Pharmacy’s Center for Diagnosis and Study of Parasitic Diseases in Timisoara, Romania. Immunoglobulin G (IgG) antibodies to *Toxocara* were determined using the Anti-*Toxocara*-ELISA Ig-G kit (Euroimmun, Lübeck, Germany) manufactured for the EUROIMMUN Analyzer I-2P. The serologic test results were interpreted based on manufacturer guidelines: <0.8 = negative, 0.8 to <1.1 = borderline, and ≥1.1 = positive [26,27]. Borderline serologic results were regarded as negative for the purposes of this investigation.

All participants were asked to fill out a self-administered questionnaire on the potential risk factors linked with *Toxocara* infection under the supervision of the principal study investigators. Demographic information, such as area of residence, gender, age, education level, and current occupation, was gathered along with personal behaviors that may have contributed to the infection (household ownership, contact with soil, dog ownership, cat ownership, consumption of raw/undercooked meat, smoking, and drinking habits). Four age groups—18–30 years old, 31–40 years old, 41–50 years old, and 51–63 years old—were established based on the age distribution of study participants.

Statistical analyses were performed using Stata 18 (StataCorp, College Station, TX, USA). Data are presented as numbers, percentages, and mean ± standard deviation (SD). An independent samples *t*-test was used to compare the mean age between seropositive and seronegative individuals. A Chi-squared (χ^2^) test was used to assess the association between *Toxocara* seroprevalence and age groups. Univariate logistic regression was employed to compare blood donors who tested positive for *Toxocara* and those who tested negative. For each univariate logistic regression conducted, we reported the crude odds ratio (cOR) along with the 95% confidence interval (95% CI). Statistical significance was set at a *p*-value < 0.05. For those factors in the univariate analysis that were shown to be significantly linked with *Toxocara* infection, stepwise multivariate logistic regression was carried out. For the multivariate model, we presented the adjusted odds ratio (aOR) along with the 95% CI. As with the univariate model, a *p*-value < 0.05 was considered statistically significant.

This study was approved by the Ethics Committee of the Victor Babes University of Medicine and Pharmacy in Timisoara (No. 4 from 8 February 2018), and informed consent was signed by all participants. The findings of their serological tests were communicated to each individual.

## 3. Results

The 1347 blood donors enrolled in the study were aged between 18 and 63 years (mean age = 33.6; SD 10.9 years), 979 (72.7%) were residents of urban areas, and 755 (56.1%) were males.

The overall seroprevalence of *Toxocara* antibodies was 29.6% (399/1347). The seropositivity was higher in individuals residing in rural areas (35.9%; 132/368) compared to those from urban areas (27.3%; 267/979) (*p* = 0.002). Furthermore, we observed that rural inhabitants have approximately a 50% increase in the odds of *Toxocara* seropositivity compared to urban inhabitants (OR = 1.49; 95% CI: 1.16–1.92) (Table 1).

We observed a higher seropositivity in males (33.3%, 251/755), compared to females (25%, 148/592) (*p* = 0.001). Moreover, males also seem to have approximately a 50% increase in the odds of *Toxocara* seropositivity compared to females (OR = 1.49, 95% CI: 1.18–1.9) (Table 1).

A significant difference between the mean age of people with anti-*Toxocara* antibodies (37 ± 11.3 years) compared to the mean age of seronegative individuals (32.3 ± 10.5 years) (*p* < 0.001) was noticed. A difference was also observed when analyzing the seroprevalence according to age groups (ꭕ^2^ = 38.2, *p* < 0.001). The percent of anti-*Toxocara* antibodies was significantly higher in individuals from 41–50 (40.1%; 109/272, *p* < 0.001) and 51–63 (44.9%; 49/109; *p* < 0.001) age groups compared to the 18–30 age group (23.6%; 143/607). The probability of people having anti-*Toxocara* antibodies was more than twice as high in those aged 41–50 years (cOR = 2.17; 95% CI: 1.6–2.95) and 51–63 years (cOR = 2.65; 95% CI: 1.74–4.04) compared to people between 18 and 30 years of age. Age was also treated as a continuous variable within the univariate logistic regression model, with each year increasing the odds of seropositivity by 4% (cOR = 1.04; 95% CI: 1.03–1.05) (Table 1).

*Toxocara* seroprevalence decreased with increasing level of education from 60% (18/30) in illiterate individuals to 29.9% (151/505) in high school graduates and 24.4% (156/638) in university graduates (*p* = 0.04). We also noticed a lower rate of seropositivity in students (21%; 61/291) compared to non-employed people (35.9%; 60/167) (*p* < 0.001) (Table 1).

In univariate analysis, household ownership (*p* < 0.001; cOR = 1.87; 95% CI: 1.46–2.4), contact with soil (*p* < 0.001; cOR = 1.9; 95% CI: 1.48–2.44), owning dogs (*p* < 0.001; OR = 1.71; 95% CI: 1.32–2.23), owning cats (*p* = 0.003; cOR = 1.59; 95% CI: 1.17–2.15), owning dog and/or cats (*p* < 0.001, cOR = 1.61; 95% CI: 1.24–2.08), and consumption of undercooked or raw poultry (*p* = 0.002; cOR = 1.7; 95% CI: 1.21–2.38) were associated with significantly higher *Toxocara* seropositivity (Table 2).

However, in a multiple logistic regression model, only a lower level of education, age, male gender, consumption of undercooked or raw poultry, and contact with soil were associated with higher *Toxocara* seropositivity (Table 3). Illiterate subjects were 4.16 times more likely than those with university degrees to have *Toxocara* antibodies. Each additional year of age increased the chance of having antibodies for *Toxocara* by 2.9%. Moreover, *Toxocara* antibodies were 1.37 times more likely to be detected in males compared to females. Individuals who consumed raw or undercooked poultry were 1.73 times more likely to have detectable antibodies compared to those who did not. In addition, subjects who came in contact with soil had 1.84 times higher odds of having antibodies compared to those who did not report soil contact.

## 4. Discussion

Human toxocariasis is considered one of the most frequent helminthic zoonotic infections in industrialized nations. The illness has been identified as a potentially major neglected infection of poverty that occurs in developed nations [18]. According to estimates, over 1.4 billion people worldwide may have been exposed to or were infected with *Toxocara* species [5].

Human toxocariasis can be diagnosed through several methods, including blood tests (detection of eosinophilia, blood count) and anatomopathological examination, as well as molecular methods that use the polymerase chain reaction (PCR) to detect larval DNA in fluid samples or tissues [28]. In an experimental research on mice, *Toxocara* DNA was found in a variety of tissues but not in the blood samples. This might be explained by the presence of the parasite in the blood only during its migration to host tissues [29].

Epidemiological surveys usually rely on serological techniques, such as ELISA and/or the Western blot method, using *Toxocara* spp. excretory–secretory antigens [28]. Serological tests are able to detect not only the infection but also the exposure to the parasite [12].

This is the first research to investigate the seroprevalence of *Toxocara* infection and its potential risk factors in healthy blood donors in Romania.

The overall prevalence of anti-*Toxocara* antibodies identified in our study was higher than that reported in blood donors from Barcelona, Spain (1%) [30]; Gualeguaychu, Argentina (10.6%) [31]; and the Slovak Republic (13.65%) [32] but lower than in Salvador-Bahia, Brazil (46.3%) [33]. Although blood donors are not entirely representative of the general population, testing for anti-*Toxocara* antibodies in this population group enables an extensive evaluation of the infection frequency and risk factors.

We identified a higher prevalence of *Toxocara* IgG antibodies in the population from a rural area. Lee et al. (2015) and Kim et al. (2014) identified higher rates of anti-*Toxocara* antibodies in asymptomatic adults and healthy healthcare examinees from Korea [34,35]. People from rural areas are expected to be exposed to contaminated soils more than people from urban areas [36]. The increasing number of stray and domesticated dogs and cats in rural regions might have boosted the rate of environmental contamination with *Toxocara* eggs. Additionally, poor hygiene practices in rural areas can also contribute to a higher occurrence of toxocariasis [36].

Our findings suggest a significantly higher seroprevalence of anti-*Toxocara* antibodies in males compared to females. Our findings align with the results published by other researchers [31,33]. This higher seroprevalence rate may be associated with behaviors and occupations that are often associated with men, like increased interaction with stray animals and soil contaminated with *Toxocara* eggs, as well as agricultural activities [28,37].

We observed that the seroprevalence of anti-*Toxocara* antibodies was higher in subjects over 40 years old, which suggests that exposure to parasites may increase with age. Similar results were reported by Mughini-Gras et al. [37] in the Netherlands and by Berrett et al. [2] in the United States. On the other hand, several studies observed that children and teenagers below the age of 20 have a higher risk of testing positive for *Toxocara* infection compared to adults. The reason could be that children are more prone to activities like playing in outdoor areas (sandboxes), being more exposed to dog and cat feces. Geophagia, sometimes practiced by children, also increases the risk of infection [14,38,39]. It is also possible that *Toxocara* exposure in Western Romania also comes from sources other than soil, given the rising seropositivity we observed with age, as previously suggested in a study conducted by Berrett et al. in 2017 in the United States [2].

In the current study, seroprevalence decreased with the increasing level of education, and was higher in non-employed people. It is known that *Toxocara* seropositivity is associated with lower educational and socioeconomic levels [40,41]. Mughini-Gras et al. also observed that seropositivity in the Netherlands tended to be higher in people with lower educational levels [37].

In the present survey, we identified household ownership and contact with soil as potential risk factors for *Toxocara* infection. *Toxocara* eggs exhibit a high resistance to a variety of environmental factors, and regular egg embryonation can occur during warm seasons. In addition, proper humidity and oxygen levels, pH, the type of soil, and the density of vegetation are additional important variables that influence not only the growth of second-stage larvae (L2) within eggs but also the resiliency and longevity of *Toxocara* eggs on the soil [42]. The estimated rate of soil contamination was higher in Romania (22%) compared to the mean estimates in Europe (18%) [5]. *Toxocara* eggs were commonly discovered in soil samples from Poland (14.9%) [43], Iran (29.2%) [44], Brazil (46%) [45], and Italy (63.6%) [46].

We identified dog, cat, dog and/or cat ownership associated with seropositivity of *Toxocara* antibodies. Over the past several decades, the world has become more urbanized, and the number of dogs and cats has significantly grown in numerous countries [5]. In Europe, infection rates among dogs and cats were estimated at 11% and 18%, respectively. Higher rates were estimated in Romania, where dogs accounted for 17% and cats for 23% prevalence [5]. Romania had the highest rate of cat ownership in the European Union in 2022, with 48% of Romanian households having at least one cat [47]. Regarding dog ownership, Romania ranked second, after Poland, with 43% of households possessing at least one dog [48]. Pet ownership is a risk factor for toxocariasis, which has been observed more commonly in households with younger dogs (rather than older dogs). The number of dogs and cats maintained in houses has grown in many countries, which might be a factor in the rising risk of infection in people [18]. The behaviors of animals (including defecation) in public spaces, such as beaches, parks, and children’s playgrounds, are likely to increase the dissemination of *Toxocara* eggs [5]. Several investigations revealed that embryonated *T. canis* and *T. cati* eggs were found on the hair of dogs and cats, respectively. This suggests that direct contact with dogs or cats may also contribute to the spread of infection [49,50,51,52]. Other studies mention that *Toxocara* eggs are very adhesive to the animals’ fur, and their removal is difficult. Therefore, it would be necessary to ingest many grams of heavily contaminated hair in order to get infected [17]. Direct egg transfer from soil to the owner’s shoes or animal paws is a potential route of *Toxocara* spp. transmission, which means that even dewormed dogs and cats might be helminth carriers [51].

We identified consumption of raw or undercooked poultry meat associated with seropositivity of anti-*Toxocara* antibodies. Consumption of raw or undercooked meat from possible paratenic homeotherm hosts, such as pigs, ostriches, chickens, cows, ducks, lambs, or rabbits, could represent a risk factor for *Toxocara* infection [13,53]. Experimental studies to assess the viability, distribution, and persistence of larvae in chickens were performed to establish if poultry consumption represents a risk factor for *Toxocara* infection in humans [54,55,56]. Due to the fact that larvae in chicken flesh have been demonstrated to be highly infectious even after longer periods of time or prolonged exposure to low temperatures, *Toxocara spp*.-infected poultry constitutes a possible health risk. Chickens raised in free-range environments are more predisposed to ingest embryonated eggs or infected paratenic hosts such as earthworms [54].

In our study, smoking and alcohol consumption were not associated with higher rates of seropositivity. Contradictory observations were made regarding the relationship between *Toxocara* and behavioral habits like smoking and drinking alcohol [36]. In Korea, in 2020, Song et al. did not find a significant association between smoking and *Toxocara* infection. However, researchers found that heavy alcohol consumption was significantly associated with *Toxocara* infection [36]. On the other hand, in a previous Korean study, Kim et al. found significantly higher rates of infection in individuals with these habits [34]. Investigators concluded that smoking and alcohol consumption may not directly transmit toxocariasis but may increase the risk of infection. Individuals, particularly men, have a propensity to consume raw cow’s meat while they smoke or drink [36], or they might consume alcohol and smoke during short breaks from working the field, without proper cleaning of their hands.

This study has some limitations. Blood donors are considered individuals in good health from certain age ranges (18–65 years) [57]. No symptoms of toxocariasis were present in those who tested positive for anti-*Toxocara* antibodies. Participation was more accessible to the urban population since the transfusion center where the donors were enrolled is located in an urban location. Even though blood donors may not represent the general population, they can be used as a study group to estimate the seroprevalence and epidemiology of *Toxocara* infection [58]. Due to the lack of pre-existing data from this region, we did not perform a Bayesian analysis, even though it could have given more detailed insights by combining prior knowledge with the collected data. Lastly, cross-sectional studies collect data at a single moment. These can show associations but not the timing of events, so they cannot confirm that an exposure came before an outcome. Because they often rely on participants’ recall, they are also vulnerable to measurement error. Thus, cross-sectional designs are useful for describing associations, not for establishing causation or event sequence [59]. In the univariate analyses, several strata were sparse (e.g., <5 observations per cell), so the cORs should be interpreted as exploratory. We used stepwise selection on a small, pre-specified covariate set; even with negligible collinearity (variance inflation factor 1.00–1.19), stepwise methods may yield unstable, selection-biased estimates.

## 5. Conclusions

The present survey offers new and important seroepidemiological information, assessing the seroprevalence and potential risk factors associated with the prevalence of *Toxocara* antibodies in blood donors. Our findings indicate that this zoonotic infection is highly prevalent in Western Romania and can be discovered in healthy, asymptomatic individuals. Seroprevalence was higher in the 51–63-year-old age group, individuals from rural areas, and males. In multiple logistic regression analysis, the rate of anti-*Toxocara* antibodies was associated with a lower level of education, age, male gender, consumption of undercooked or raw poultry, and contact with soil. Further research on the general population is needed to estimate the prevalence of human toxocariasis in Romania.

Toxocariasis prevention is a significant challenge due to the various infection origins and transmission pathways of *Toxocara* spp., which are currently poorly understood. Preventive methods like washing hands after contact with soil or pets and avoiding consumption of undercooked meat could help in reducing exposure to *Toxocara* infection. Moreover, constant deworming of pets and limitation of free-roaming dogs and cats, and cleaning up feces from the soil should reduce the spread of parasitic eggs.

## Figures and Tables

**Table 1:** Univariate analysis of the demographic data of blood donors from Western Romania, stratified by *Toxocara* seropositivity, ascertained by questionnaire.

Variables	Number of Participants Without Detectable Anti-*Toxocara* Antibodies (%)	Number of Participants with Detectable Anti-*Toxocara* Antibodies (%)	*p*-Value	cOR (95%CI)
**Area of residence**				1.49 (1.16–1.92)
** ** **Urban**	712 (72.7)	267 (27.3)	0.002	
** ** **Rural**	236 (64.1)	132 (35.9)	Ref.	
** ** **Gender**				1.49 (1.18–1.9)
** ** **Female**	444 (75)	148 (25)	0.001	
** ** **Male**	504 (66.7)	251 (33.3)	Ref.	
Age *	-	-	<0.001	1.04 (1.03–1.05)
**Age groups (years)**				
** ** **18–30**	464 (76.4)	143 (23.6)	Ref.	-
** ** **31–40**	261 (72.7)	98 (27.3)	0.19	1.21 (0.9–1.64)
** ** **41–50**	163 (59.9)	109 (40.1)	<0.001	2.17 (1.6–2.95)
** ** **51–63**	60 (55.1)	49 (44.9)	<0.001	2.65 (1.74–4.04)
**Education level**				
** ** **Illiterate**	12 (40)	18 (60)	<0.001	4.63 (2.18–9.84)
** ** **Gymnasium**	100 (57.5)	74 (42.5)	<0.001	2.29 (1.61–3.25)
** ** **Highschool**	354 (70.1)	151 (29.9)	0.04	1.32 (1.01–1.71)
** ** **University**	482 (75.6)	156 (24.4)	Ref.	-
** ** **Occupational status**				
** ** **Non-employed**	107 (64.1)	60 (35.9)	Ref.	-
** ** **Employed**	596 (68.6)	273 (31.4)	0.25	0.82 (0.58–1.16)
** ** **Student**	230 (79)	61 (21)	<0.001	0.47 (0.3–0.72)
** ** **Retiree**	15 (75)	5 (5)	0.33	0.59 (0.21–1.71)

*p*-value, probability value; cOR, crude odds ratio; Ref. = reference; * = age was analyzed as a continuous variable; the cOR reflects the change per year; CI, confidence interval.

**Table 2:** Univariate analysis of the risk factors associated with *Toxocara* seropositivity in blood donors from Western Romania.

**Variables**	**Number of Participants Without Detectable Anti-*Toxocara* Antibodies (%)**	**Number of Participants with Detectable Anti-*Toxocara* Antibodies (%)**	***p*** **-Value**	**cOR (95%CI)**
**Household ownership**				
** ** **No**	708 (74.4)	244 (25.6)	Ref.	
** ** **Yes**	240 (60.8)	155 (39.2)	<0.001	1.87 (1.46–2.4)
**Contact with soil**				
** ** **No**	707 (74.5)	242 (25.5)	Ref.	
** ** **Yes**	241 (60.6)	157 (39.5)	<0.001	1.9 (1.48–2.44)
**Working in agriculture**				
** ** **No**	946 (70.4)	397 (29.6)	Ref.	
** ** **Yes**	2 (50)	2 (50)	0.39	2.38 (0.33–16.98)
**Owning dogs**				
** ** **No**	747 (73.2)	273 (26.8)	Ref.	
** ** **Yes**	201 (61.5)	126 (38.5)	<0.001	1.71 (1.32–2.23)
**Owning cats**				
** ** **No**	815 (72)	317 (28)	Ref.	
** ** **Yes**	133 (61.9)	82 (38.1)	0.003	1.59 (1.17–2.15)
**Owning cat or/and dog**				
** ** **No**	723 (73.1)	266 (26.9)	Ref.	
** ** **Yes**	225 (62.9)	133 (37.1)	<0.001	1.61 (1.24–2.08)
**Consumption of raw meat**				
** ** **No**	551 (70.8)	227 (29.2)	Ref.	
** ** **Yes**	397 (69.8)	172 (30.2)	0.68	1.05 (0.83–1.33)
**Consumption of raw/undercooked poultry**				
** ** **No**	849 (71.8)	333 (28.2)	Ref.	
** ** **Yes**	99 (60)	66 (40)	0.002	1.7 (1.21–2.38)
**Consumption of raw/undercooked pork**				
** ** **No**	679 (71.9)	265 (28.1)	Ref.	
** ** **Yes**	269 (66.8)	134 (33.2)	0.06	1.27 (0.99–1.64)
**Consumption of raw/undercooked wild boar**				
** ** **No**	721 (70.6)	300 (29.4)	Ref.	
** ** **Yes**	227 (69.6)	99 (30.4)	0.73	1.05 (0.8–1.38)
**Smoking**				
** ** **No**	642 (69.9)	276 (30.1)	Ref.	
** ** **Yes**	306 (71.3)	123 (28.7)	0.6	0.94 (0.73–1.2)
**Alcohol**				
** ** **No**	311 (67.5)	150 (32.5)	Ref.	
** ** **Yes**	637 (71.9)	249 (28.1)	0.09	0.81 (0.64–1.03)

*p*-value, probability value; cOR, crude odds ratio; Ref. = reference; CI, confidence interval.

**Table 3:** Risk factors associated with *Toxocara* seroprevalence in blood donors from Western Romania (stepwise multivariate logistic regression).

Variable	aOR	95% CI	*p*-Value
**Illiterate**	4.16	1.93–8.96	<0.001
**Gymnasium**	1.69	1.16–2.45	0.006
**Highschool**	1.25	0.95–1.64	0.106
**University**	Ref.		
**Age (as a continuous variable)**	1.029	1.02–1.04	<0.001
**Male (Ref.: females)**	1.37	1.07–1.76	0.013
**Consumption of raw or undercooked poultry (Ref.: no)**	1.73	1.22–2.45	0.002
**Contact with soil (Ref.: no)**	1.84	1.42–2.38	<0.001

Ref. = reference; *p*-value, probability value; aOR, adjusted odds ratio; CI, confidence interval.

## Data Availability

All relevant data are contained within the article.

## Abbreviations

The following abbreviations are used in this manuscript:

ELISAs	Enzyme-linked immunosorbent assays
*T. canis*	*Toxocara canis*
*T. cati*	*Toxocara cati*
IgG	Immunoglobulin G
WB	Western blot
